# Supplementary material for: Carnivore coexistence without competition: giant otters are more nocturnal around dens than sympatric neotropical otters
Source: PeerJ. 2024 Apr 5;12:e17244. doi: 10.7717/peerj.17244 (PMC11000638; doi:10.7717/peerj.17244)
Supplement: Supplemental Information 1 [file peerj-12-17244-s001.docx]

Supplemental material

S1 General overview of otter dens.

| Den 1 | Den 2 |
| --- | --- |
| 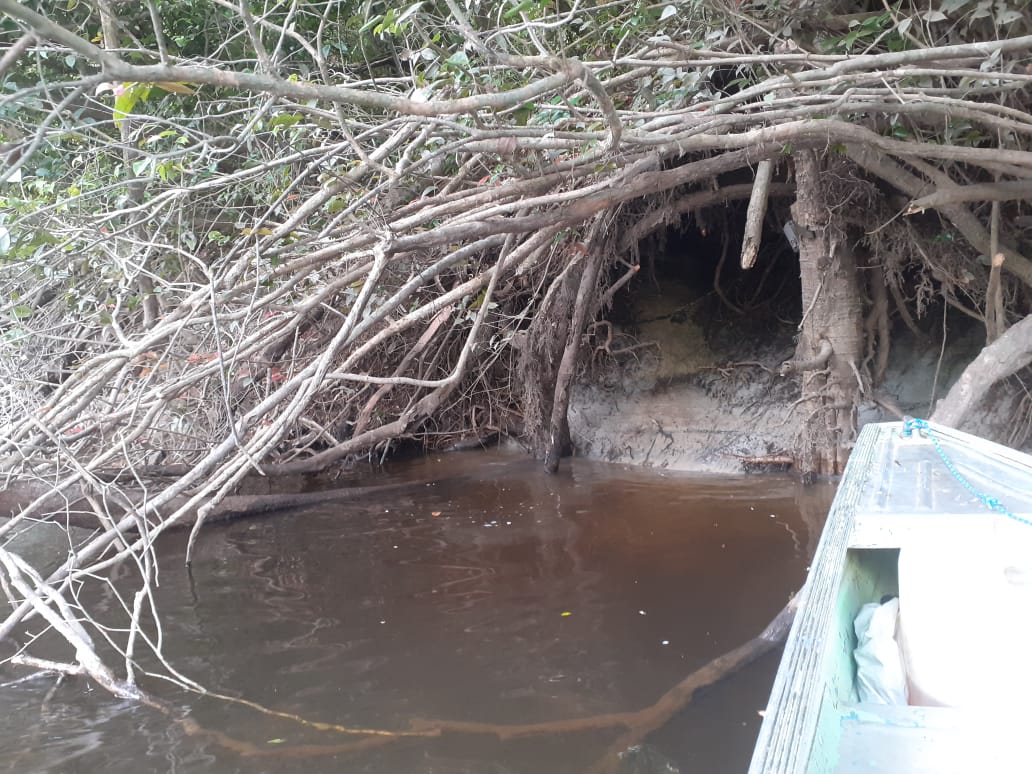 | 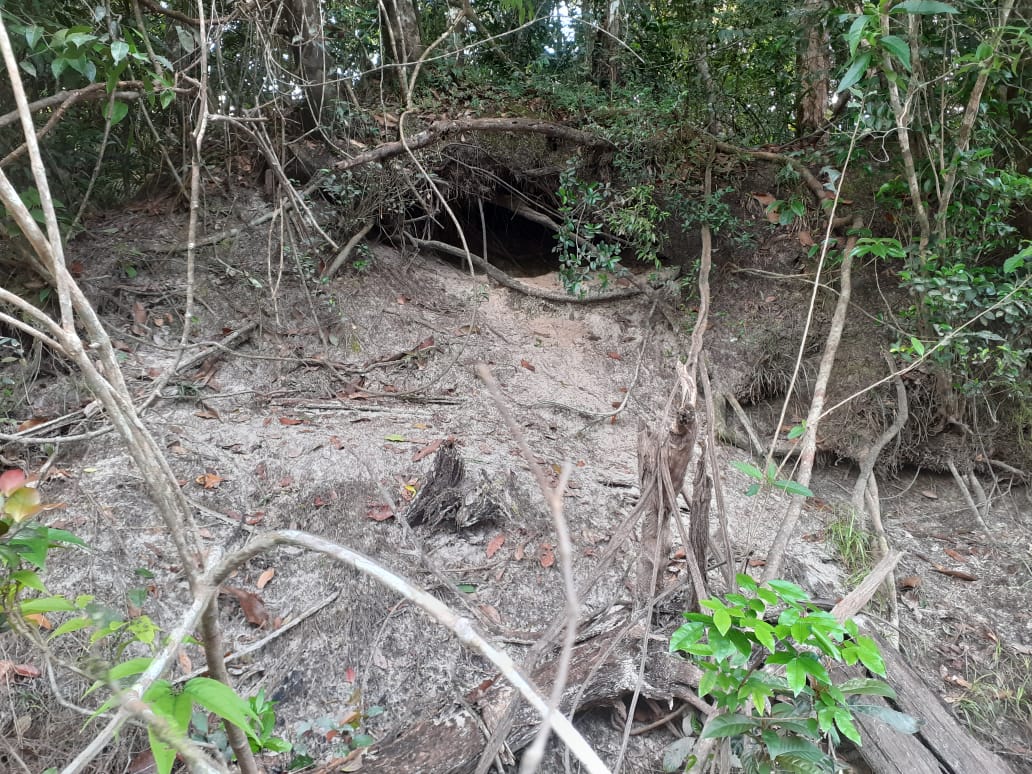 |
| Den 3 | Den 4 |
| 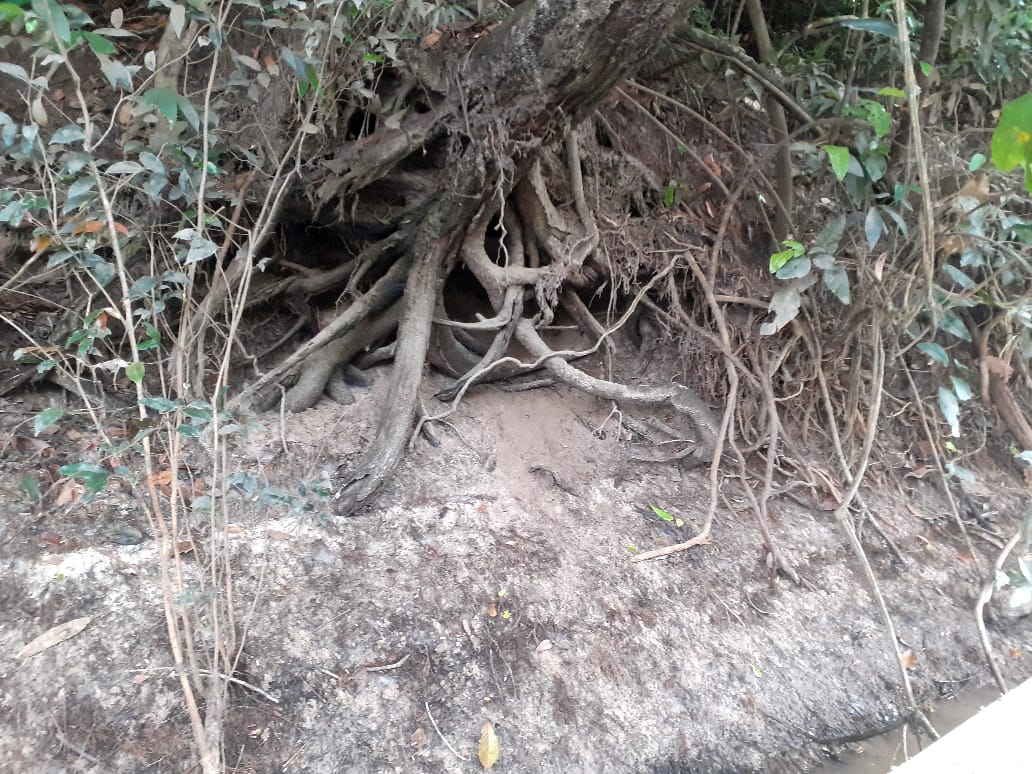 | 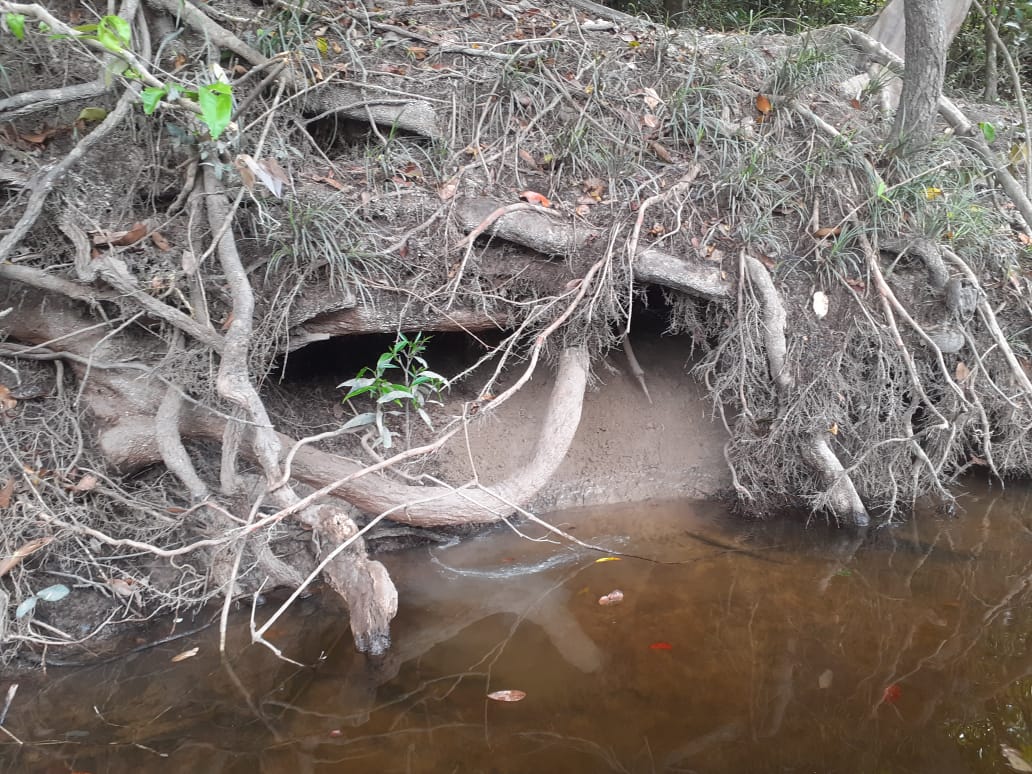 |
| Den 5 |  |
| 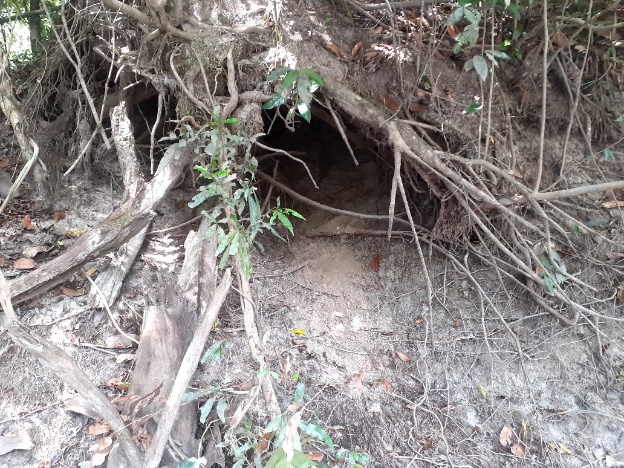 |  |

S2 Den characteristics, survey effort and otter detections

Table S2. Effort and detections. Total of all photos per species and independent photos per 10 trap days in parenthesis.

|  |  | |  | | | Den | |  | |  | | Totals |
| --- | --- | --- | --- | --- | --- | --- | --- | --- | --- | --- | --- | --- |
|  | D1 | | D2 | | | D3 | | D4 | | D5 | |  |
|  |  | |  | |  | | |  | |  | |  |
| Km^a^ | 0 | | 1.8 | | 3.1 | | | 4.0 | | 12.8 | |  |
| Entrance dimensions (height x width, cm) | 90 x 160 | | 60 x 160 | | 70 x 80 | | | 60 x 155 | | 90 X 75 | |  |
| Distance to water (cm)^b^ | 45 | | 330 | | 140 | | | 60 | | 160 | |  |
| Distance to camera (cm) | 95 | | 193 | | 166 | | | 178 | | 102 | |  |
| CT days | 88 | | 77 | | 77 | | | 77 | | 77 | | 396 |
| Main uses | Rearing/ resting | | Temporary resting | | Temporary resting | | | Temporary resting | | Temporary resting | |  |
| Scent marking (giant otter / neotropical otter) | Yes / NA | | Yes / NA | | NA / Yes | | | No / Yes | | NA / Yes | |  |
| Number of individuals (giant otter / neotropical otter) ^c^ | 7/0 | | 3/0 | | 0/2 | | | 2/2 | | 0/2 | | 7/2 |
| **Species^c^** |  |  |  |  |  | |  |  |  |  |  |  |
| Giant otter | 1668 | (10.0) | 53 | (0.7) | 0 | |  | 39 | (0.9) | 0 |  | 1760 (2.53) |
| Neotropical otter | 0 |  | 0 |  | 53 | | (2.0) | 129 | (3.5) | 114 | (3.1) | 296 (1.67) |
|  |  |  |  |  |  | |  |  |  |  |  |  |
|  |  |  |  |  |  | |  |  |  |  |  |  |
|  |  |  |  |  |  | |  |  |  |  |  |  |

^a^ Upstream distance along river from Den 1 (kilometers).

^b^ Distance from den entrance to rivers edge (centimeters). Measured along the ground to reflect distance that an otter would walk.

^c^ Number of individuals identified

^d^ Total of all photos per species and independent photos per 10 trap days in parenthesis.

S3 Den events

The difference in time between consecutive photos was used to determine distinct den activity events. While previous studies have established events using an apparently arbitrary value of 10 minutes (Leuchtenberger et al. 2014), we used the distribution of time between consecutive photos to obtain the value. This classification came from the complete series of photographs where den events were established based on timing and could include any combination of entering, leaving and/or activity in front of the den. As individuals and groups may trigger repeated photos the distribution of time between consecutive photos was calculated at each den. The event classification did not depend on individual identification and did not require a proportion of the group to be photographed. This was chosen to provide a standardized comparison between the species. Additionally, as individuals can repeatedly enter and leave this also prevents artificial separation of events that do not reflect the overall activity. For both species most consecutive photos were taken within an hour. As giant otters were most frequently recorded, we used data from this species to establish a 22.6 minute threshold for distinct den events. This value corresponded to the 90% percentile of all consecutive difference values for giant otters.


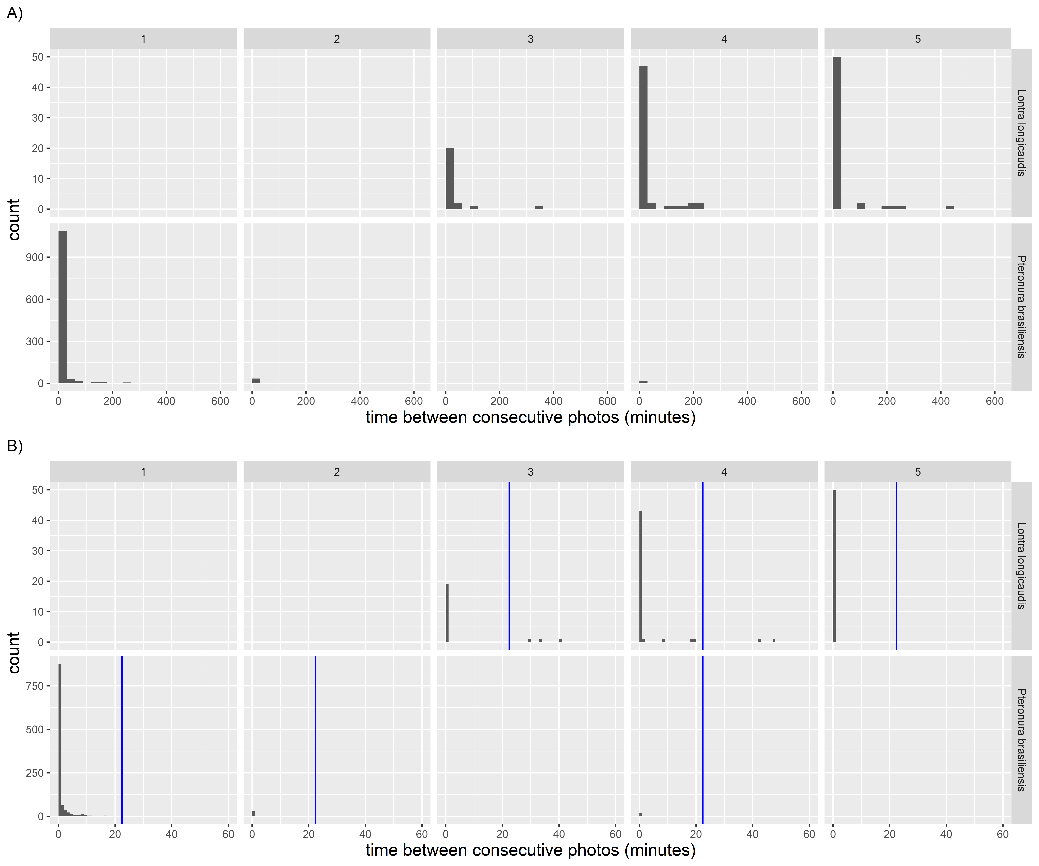


Figure S1. Time between consecutive photos. Time differences for all (A) and (B) subset of photos within 60 minutes. Counts of photos grouped by 30 and one minute bins for (A) and (B) respectively. Solid blue vertical line (B) is the 22.6-minute threshold used to identify den events.

S4 Adult otters at the same den. Photos of (A) adult giant otters and (B) neotropical otters at Den 4.

| 1. Giant otters. |
| --- |
| 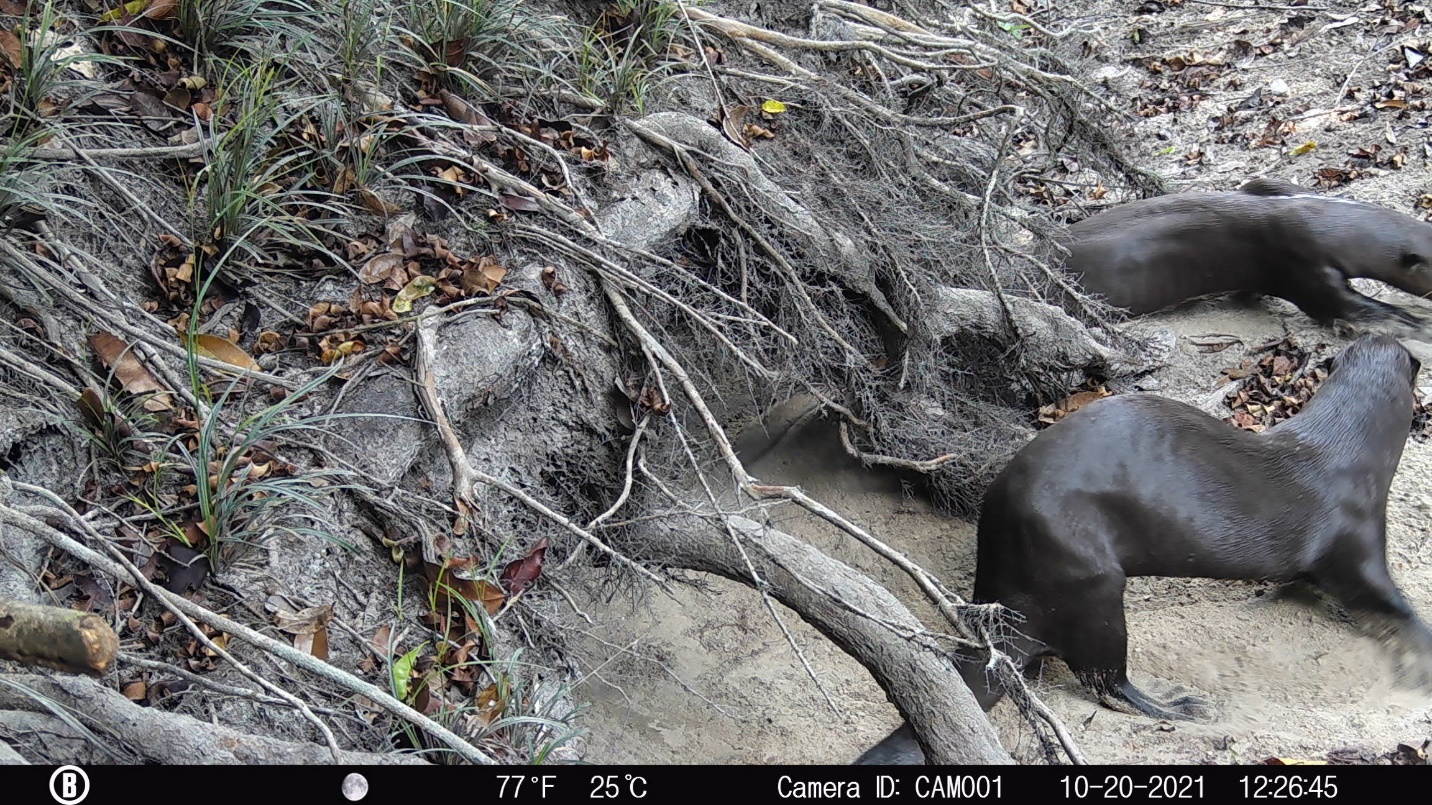 |
| 1. Neotropical otters |
| 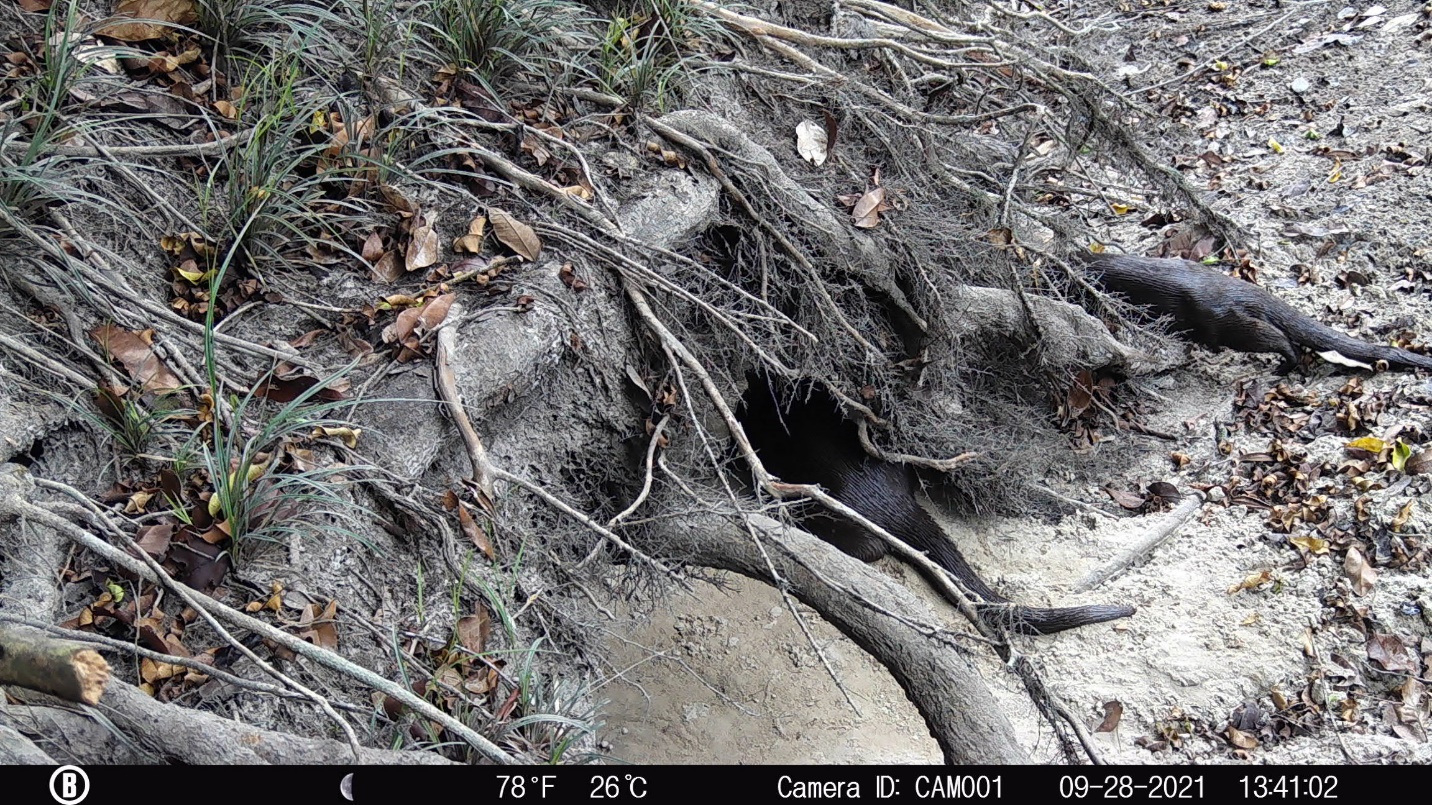 |

References

Leuchtenberger C, Zucco CA, Ribas C, Magnusson W, and Mourão G. 2014. Activity patterns of giant otters recorded by telemetry and camera traps. *Ethology Ecology & Evolution* 26:19-28. <https://doi.org/10.1080/03949370.2013.821673>
